# Association Between Serum Furin and Fasting Glucose: A Cross-Sectional Study in Chinese Adults

**DOI:** 10.3389/fendo.2021.781890

**Published:** 2022-01-03

**Authors:** Yan He, Hanyun Zhu, Min Zhang, Jing Li, Shengqi Ma, Yin Lu, Linan Chen, Mingzhi Zhang, Hao Peng

**Affiliations:** ^1^ Department of Epidemiology, School of Public Health, Medical College of Soochow University, Suzhou, China; ^2^ Department of Biostatistics, School of Public Health, Medical College of Soochow University, Suzhou, China; ^3^ Central Office, Suzhou National New and Hi-Tech Industrial Development Zone Center for Disease Control and Prevention, Suzhou, China; ^4^ Jiangsu Key Laboratory of Preventive and Translational Medicine for Geriatric Diseases, Suzhou, China

**Keywords:** furin, diabetes, fasting plasma glucose, Chinese, cross-sectional study, risk factor

## Abstract

**Background:**

Furin has been associated with glucose metabolic phenotypes in small sampled clinical studies. However, this association has not yet been studied in Chinese. Here, we aimed to examine the association between serum furin and fasting glucose in Chinese adults.

**Methods:**

Serum furin and fasting plasma glucose were assayed for 2,172 participants (mean aged 53 years, 38% men) in the Gusu cohort. A median regression model was applied to examine the association between serum furin and fasting glucose, adjusting for age, sex, education level, cigarette smoking, alcohol drinking, obesity, blood pressure, and lipids. To facilitate data interpretation, the association between serum furin and prevalent diabetes was also examined.

**Results:**

Serum furin was negatively associated with fasting glucose (β=-0.18, *P*<0.001 for log-furin). In participants with diabetes, serum furin was significantly lower than those with normal glucose (median: 0.90 ng/mL *vs.* 1.05 ng/mL, *P*=0.001). Compared with participants in the highest quartile of serum furin, those in the lowest quartile had 42% and 80% increased risk of prevalent prediabetes (OR=1.42, 95%CI: 1.05-1.92, *P*=0.023) and diabetes (OR=1.80, 95%CI: 1.13-2.91, *P*=0.015), respectively.

**Conclusions:**

Serum furin was negatively associated with prediabetes and diabetes in Chinese adults. Our findings suggest that serum furin may be a risk factor or a biomarker of diabetes.

## Introduction

Furin, ubiquitously expressed in all mammalian tissues and cells, is a member of the proprotein convertase subtilisin/Kexin (PCSK) family (1). Accumulating evidence has demonstrated that furin may be implicated in the process of glucose metabolism by converting numerous protein and peptide precursors into their bioactive forms. For example, cell-based studies found that furin could activate the precursors of insulin and its receptor (2, 3) and regulate the proliferation and differentiation of pancreatic β-cells which determines the secretion of insulin (4, 5). Animal experiments found that knockout of the *furin* gene in β-cells resulted in glucose intolerance in mice (6). In humans, polymorphisms in the *FURIN* gene have been associated with metabolic syndrome (7), hypertension (8), and coronary artery disease (9). These findings suggest a potential role of furin in glucose metabolism. In fact, the levels of furin in the circulation have been associated with diabetes (10) and some relative phenotypes, such as obesity (11), metabolic syndrome (12), and diabetic cardiovascular disease (13). However, these results were largely derived from populations with European ancestry. It’s not clear whether circulating furin could be associated with glucose metabolism in Chinese who have a different genetic background and risk profiles of diabetes in comparison to European populations. In Chinese adults, we previously found that serum furin was associated with obesity (14) and hypertension (15). Whether serum furin is associated with diabetes has not been studied in Chinese. Therefore, we aimed to examine the association between serum furin and fasting plasma glucose in Chinese adults.

## Methods

### Participants

The Gusu cohort is a prospective study aiming to identify new risk factors for cardiovascular disease (CVD) in Chinese adults. The study design, survey methods, and laboratory measurements have been detailed previously (16). In brief, 2,498 community members aged over 30 years and free of CVD and chronic kidney disease were enrolled in the baseline examination in 2010. After excluding participants with missing data on serum furin (n=326), 2172 participants were finally included in the current analysis. The protocols of the current study were approved by the Soochow University Ethics Committee (Approval No. SUDA20200601H02). Written informed consent was obtained from all study participants.

### Measurement of Serum Furin

Using the -80°C stored serums obtained in the baseline examination, furin concentrations were measured using commercial ELISA kits (Catalog: DL-FUR-Hu; DLDEVELOP, Wuxi, China) according to standard protocols as previously described (15). All the samples were processed in a duplicate assay. A standard curve was constructed and from which furin concentrations of unknown samples were determined.

### Measurement of Fasting Plasma Glucose

Blood samples were obtained by venipuncture in the morning after a requested overnight fast (at least 8 h). Within 4 hours after venipuncture, fasting plasma glucose (FPG) was analyzed enzymatically on a Hitachi 7020 automatic biochemical analyzer using commercial reagents (Kangxiang Medical Appliances, Shanghai, PR of China). Intra- and inter-assay coefficients of variation were less than 2% and 4%, respectively. Diabetes was defined as the presence of one of the following: (a) a self-reported previous diagnosis by health care professionals and current use of either insulin or oral hypoglycemic medication, and (b) an FPG level of 7.0 mmol/L or higher (17). Prediabetes was defined as an FPG level between 5.6 and 6.9 mmol/L without self-reported physician‐diagnosed diabetes.

### Assessment of Conventional Risk Factors

Data on demographic information, lifestyle risk factors, and personal medical history were collected with standard questionnaires in the Chinese language administered by trained staff. Current smoking was defined as having smoked at least 100 cigarettes in the entire lifetime, smoke cigarettes regularly, and smoke currently. Current drinking was defined as having consumed alcohol ≥ 12 times in the past year and drinking currently. Education level was estimated as years a participant stays in the education system. Body mass index (BMI) was calculated as weight in kilograms divided by the square of the subject’s height in squared meters. Three consecutive sitting blood pressure measurements (3 min between each) were taken by trained staff, using a standard mercury sphygmomanometer, according to standard protocol (18), after the subjects had been resting for at least 5 min. The first and fifth Korotkoff sounds were recorded as systolic blood pressure (SBP) and diastolic blood pressure (DBP), respectively. The mean of the three records was used in the analysis. Total cholesterol (TC), triglycerides (TG), high-density lipoprotein cholesterol (HDL-C), and low-density lipoprotein cholesterol (LDL-C) were examined for all participants.

### Statistical Analysis

Baseline characteristics of study participants were presented according to quartiles of serum furin. Base-10 logarithmic transformation was applied to maximal normal distribution of serum furin and the generated values (log-furin) were used in downstream analyses. To examine the association between serum furin and FPG, we constructed a median regression model in which FPG was the dependent variable and serum furin (continuous log-furin or categorical furin in quartiles) was the independent variable, adjusting for age, sex, education level, cigarette smoking, alcohol drinking, BMI, SBP, LDL-C, and HDL-C. Median regression was used here to account for the skewed distribution data of FPG. To facilitate data interpretation, we further examined the association between serum furin and prevalent diabetes by constructing a logistic regression model in which prevalent diabetes (yes/no) was the dependent variable and serum furin (log-furin or furin quartiles) was the independent variable, adjusting for the confounding factors listed above. The association between serum furin and prediabetes was similarly examined. All statistical analyses were conducted using SAS statistical software (version 9.1, Cary, NC). A two-tailed *P* value less than 0.05 was considered statistically significant.

## Results

### Baseline Characteristics

There were 2,172 participants (823 men and 1349 women) with a mean age of 53.2 years included in the present study. Of them, 198 (9.12%) participants including 85 patients under hypoglycemic treatments were diagnosed with prevalent diabetes. Their baseline characteristics according to quartiles of serum furin are shown in **Table 1**. Compared with participants with a higher level of serum furin, those with a lower level of serum furin were more likely to be older, current smokers, current drinkers and had higher levels of BMI, SBP, DBP, and FPG (all *P*<0.05).

**Table 1:** Baseline characteristics of study participants according to serum furin levels (N = 2172).

Characteristics	Serum furin, ng/mL				*P*-value for trend
	Quartile 1 (~0.59)	Quartile 2 (0.60~1.01)	Quartile 3 (1.02~1.75)	Quartile 4 (1.76~)	
No. of participants	544	543	543	542	
Age, years, mean ± SD	54.37 ± 9.51	53.49 ± 9.51	51.90 ± 9.52	51.58 ± 9.57	<0.001
Sex, men (%)	254 (46.69)	208 (38.31)	180 (33.15)	181 (33.39)	<0.001
Current smoking, n (%)	146 (26.84)	122 (22.47)	96 (17.68)	121 (22.32)	0.022
Current drinking, n (%)	128 (23.53)	103 (18.97)	88 (16.21)	76 (14.02)	<0.001
Education level, years, mean ± SD	7.18 ± 3.31	7.32 ± 3.14	7.04 ± 3.26	6.83 ± 3.21	0.033
BMI, kg/m^2^, mean ± SD	24.91 ± 3.70	25.10 ± 3.46	24.78 ± 3.46	24.51 ± 3.83	0.031
SBP, mmHg, mean ± SD	132.9 ± 15.5	131.1 ± 14.3	129.1 ± 15.8	128.3 ± 20.1	<0.001
DBP, mmHg, mean ± SD	86.3 ± 7.6	85.6 ± 8.1	84.4 ± 8.9	84.0 ± 11.3	<0.001
Fasting glucose, mmol/L	5.3 (4.8-5.8)	5.2 (4.7-5.7)	5.1 (4.7-5.6)	5.0 (4.6-5.6)	0.002
Total cholesterol, mmol/L	5.05 (4.50-5.63)	5.10 (4.56-5.74)	5.09 (4.53-5.77)	5.11 (4.55-5.68)	0.154
Triglycerides, mmol/L	1.09 (0.79-1.50)	1.19 (0.82-1.70)	1.08 (0.77-1.68)	1.11 (0.75-1.65)	0.428
LDL cholesterol, mmol/L	2.96 (2.56-3.50)	2.97 (2.51-3.46)	2.93 (2.50-3.37)	2.92 (2.48-3.39)	0.164
HDL cholesterol, mmol/L	1.44 (1.24-1.71)	1.43 (1.19-1.70)	1.50 (1.26-1.75)	1.45 (1.22-1.72)	0.612

Results are expressed as median (interquartile range) unless otherwise noted. P-values for trend were tested by linear regression model for continuous variables and chi-square trend test for categorical variables.

BMI, body mass index; SBP, systolic blood pressure; DBP, diastolic blood pressure; LDL, low-density lipoprotein; HDL, high-density lipoprotein.

### Levels of Serum Furin in Participants With Different Statuses of Glucose Metabolism

**Figure 1:**
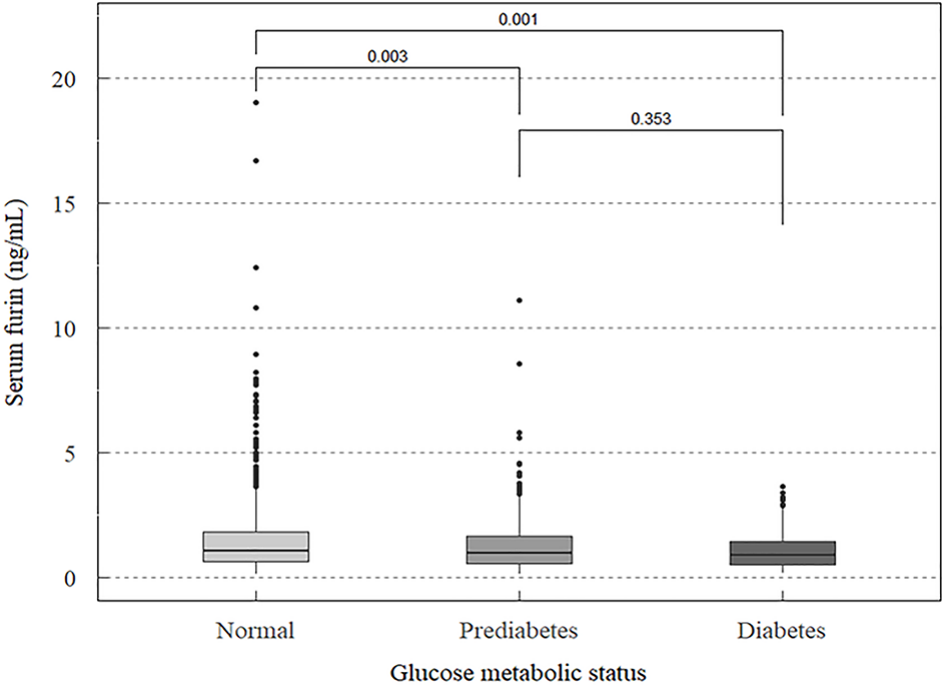
Median levels of serum furin in participants with different statuses of glucose metabolism.

Intra- and inter-assay coefficients of variation of the measurements of serum furin were less than 10% and 12%, respectively. Compared to participants with a normal FPG, the median levels of serum furin was significantly lower in those with prediabetes (median: 0.96 ng/mL *vs.* 1.05 ng/mL, *P*=0.003) and diabetes (median: 0.90 ng/mL *vs.* 1.05 ng/mL, *P*=0.001) (**Figure 1**). We did not find any significant difference in serum furin levels between participants with prediabetes and diabetes (*P*=0.353).

### Association Between Serum Furin and Glucose Metabolic Status

After adjusting for age, sex, education level, cigarette smoking, alcohol drinking, BMI, SBP, LDL-C, and HDL-C, serum furin was negatively associated with FPG (β=-0.18, *P*<0.001 for log-furin, **Table 2**). Compared with participants at the highest quartile of serum furin, those at the lowest quartile had a median of 0.19 mmol/L higher FPG (P<0.001). The regression with prevalent prediabetes and diabetes revealed that a higher level of serum furin seemed to be nominally associated with a lower risk of having prediabetes (OR=0.72, *P*=0.051) and diabetes (OR=0.62, *P*=0.052) (**Table 3**). Compared with participants at the highest quartile of serum furin, those at the lowest quartile had 42% and 80% increased risk of having prediabetes (*P*=0.023) and diabetes (*P*=0.015), respectively.

**Table 2:** Association between serum furin and fasting plasma glucose.

serum furin(ng/mL)	Un-adjusted		Adjusted^*^	
	β (SE)	*P*-value	β (SE)	*P*-value
log-furin	-0.27 (0.06)	<0.001	-0.18 (0.05)	<0.001
Categorical				
Quartile 4	ref		ref	
Quarntile 3	0.10 (0.05)	0.053	0.08 (0.04)	0.062
Quarntile 2	0.20 (0.05)	<0.001	0.04 (0.05)	0.393
Quarntile 1	0.30 (0.05)	<0.001	0.19 (0.04)	<0.001

*Adjusting for age, sex, education level, cigarette smoking, alcohol consumption, SBP, BMI, LDL, and HDL.

**Table 3:** Associations between serum furin and prevalent prediabetes and diabetes.

serum furin(ng/mL)	Prediabetes			Diabetes		
	Cases	OR (95%CI)*	*P*-value	Cases	OR (95%CI)*	*P*-value
log-furin	474	0.72 (0.52-1.00)	0.051	198	0.62 (0.38-1.00)	0.052
Categorical						
Quartile 4	108	ref		33	ref	
Quartile 3	107	1.05 (0.76-1.43)	0.779	55	1.74 (1.08-2.82)	0.024
Quartile 2	114	0.98 (0.72-1.34)	0.910	48	1.28 (0.79-2.10)	0.315
Quartile 1	145	1.42 (1.05-1.92)	0.023	62	1.80 (1.13-2.91)	0.015

*Adjusting for age, sex, education level, cigarette smoking, alcohol consumption, SBP, BMI, LDL, and HDL.

## Discussion

We are the first to report an association between serum furin and fasting glucose in Chinese adults in a cross-sectional study including a relatively large sample of participants in the Gusu cohort. Participants with a lower level of serum furin were more likely to have prevalent prediabetes and diabetes. This association was independent of behavioral and metabolic factors. Our findings indicated that furin may participate in glucose metabolism through mechanisms beyond metabolic factors. Furin deficiency may be a marker or even a potential risk factor for diabetes.

The identified association between serum furin and glucose metabolism found in our study has also been suggested by other studies. For example, β cell-specific *furin* gene knockout mice developed glucose intolerance and had smaller islets with lower insulin content than wild controls (6). In humans, polymorphisms of the *FURIN* gene encoding furin protein have been associated with some diabetic complications, such as metabolic syndrome (7), hypertension (8), and coronary artery disease (9). Further, furin protein in circulation has also been studied by some clinical studies. For example, a case-control study including 25 diabetic patients with complications, 25 diabetic patients without complications, and 25 healthy controls found that serum furin was significantly associated with diabetes complicated with cardiovascular disease (13). A cross-sectional study including 138 participants found that furin was significantly associated with metabolic syndrome (12). A prospective study including 4,678 participants reported an association of serum furin with incident diabetes among Swedish in the Malmö Diet and Cancer Study (10). However, these results were mainly generated from White populations whose genetic background and diabetes risk profiles differ from Chinese. Our study is the first to examine the association between serum furin and diabetes in Chinese adults. Nevertheless, we found an inconsistent result that a lower level of serum furin was associated with a higher risk of prevalent diabetes. Different genetic backgrounds of study participants may explain the discrepancy. Notably, basic studies demonstrated that furin deficiency caused by gene knockout could result in glucose intolerance (6). This finding may indicate a feedback adjustment mechanism that increased glucose in diabetic patients stimulates secretion of furin in feedback to facilitate activation of insulin and its receptors. Therefore, researchers may observe a positive association between serum furin and diabetes in populations as listed above. These results appeared to indicate that the relation between serum furin and diabetes in populations is more complex than that in cell and animal experiments. As a wide proprotein convertase, furin acts as an upstream regulator of the glucose metabolism system, such as the natriuretic peptides system. This system plays a critical role in maintaining blood glucose balance and blood pressure through BNP which was activated by furin. As a result, furin expression and excretion may be upregulated in compensatory for high-risk individuals of diabetes, e.g., hypertensive and obesity patients. Also, our group previously found that serum furin was also significantly associated with obesity and hypertension that share many risk factors and molecular mechanisms with diabetes (14, 15). All these findings, even conflicting, together with ours, suggest that furin may participate in glucose metabolism and therefore could be a therapeutic target for diabetes.

In addition to population evidence, the possible mechanisms underlying the association between serum furin and diabetes could deepen our understanding of the role of furin in diabetes. Furin, which belongs to the PCSK family and converts numerous proteins and peptide precursors into their biologically active forms, has been demonstrated to play an important role in glucose metabolism and several relative processes associated with diabetes, such as insulin resistance. One possible mechanism could be that furin deficiency influences the maturation of insulin receptors (2, 19) which is critical for the maintenance of glucose homeostasis. The second possible mechanism may be the involvement of furin in the proliferation and differentiation of pancreatic β-cells (5) where glucose metabolism mainly occurs. Another possible mechanism might be related to the role of furin in the activation of B-type natriuretic peptides (20, 21) which is one key component of the natriuretic peptides system. This system has been demonstrated to play an integral role in glucose metabolism and participate in the development of diabetes (20, 22, 23).

To the best of our knowledge, our study is the first to investigate the association between serum furin and diabetes in Chinese adults. The strengths of this study include careful and systemic analyses of the association between serum furin and glucose metabolic states including prediabetes and diabetes and comprehensive adjustments of many conventional risk factors including behavioral and metabolic factors. However, our study also has several limitations that deserve clarification. First, the cross-sectional study designs prevented a causal inference. However, our results showed that serum furin was not only associated with diabetes but also correlated with prediabetes. This finding increased the probability that furin deficiency may be a risk factor for diabetes. Nevertheless, the causality between serum furin and diabetes is not established and needs further evidence from clinical trials. Second, our study population was comprised of ethnic Han individuals only, whether the results can be generalizable to other racial/ethnic groups is not clear.

## Conclusion

In conclusion, our study demonstrated that serum furin was negatively associated with prediabetes and diabetes in Chinese adults. These results suggested that furin might play a potential role in glucose metabolism and the deficiency of furin might serve as a risk factor or biomarker for diabetes. However, the causal association between furin and diabetes still needs more evidence and the underlying molecular mechanisms still need further investigations.

## Data Availability Statement

The raw data supporting the conclusions of this article will be made available by the authors, without undue reservation.

## Ethics Statement

The studies involving human participants were reviewed and approved by Soochow University Ethics Committee. The patients/participants provided their written informed consent to participate in this study.

## Author Contributions

YH, MZZ, and HP conceived and designed the study. HZ and MZ analyzed and interpreted the data. YH and HZ drafted the paper. MZ, YH, JL, YL, LC, and SM collected the data. MZZ and HP revised and gave the final approval of the version to be published, and all authors agreed to be accountable for all aspects of the work.

## Funding

This study was supported by the National Natural Science Foundation of China (NO. 82173596, 81903384, and 81872690), the Natural Science Foundation of Jiangsu Province (NO. BK20180841), the Suzhou Municipal Science and Technology Bureau (NO. SS201853, SKJY2021040, and SYS2020091), and a Project of the Priority Academic Program Development of Jiangsu Higher Education Institutions.

## Conflict of Interest

The authors declare that the research was conducted in the absence of any commercial or financial relationships that could be construed as a potential conflict of interest.

## Publisher’s Note

All claims expressed in this article are solely those of the authors and do not necessarily represent those of their affiliated organizations, or those of the publisher, the editors and the reviewers. Any product that may be evaluated in this article, or claim that may be made by its manufacturer, is not guaranteed or endorsed by the publisher.
